# Human endogenous retroviruses and the inflammatory response: A vicious circle associated with health and illness

**DOI:** 10.3389/fimmu.2022.1057791

**Published:** 2022-11-23

**Authors:** Sara Coelho Rangel, Michelly Damasceno da Silva, Amanda Lopes da Silva, Juliana de Melo Batista dos Santos, Lucas Melo Neves, Ana Pedrosa, Fernanda Monteiro Rodrigues, Caio dos Santos Trettel, Guilherme Eustáquio Furtado, Marcelo Paes de Barros, André Luis Lacerda Bachi, Camila Malta Romano, Luiz Henrique Da Silva Nali

**Affiliations:** ^1^ UNISA Research Center, Universidade Santo Amaro, Post-Graduation in Health Sciences, São Paulo, Brazil; ^2^ Laboratório de Virologia, Instituto de Medicina Tropical de São Paulo, Universidade de São Paulo, São Paulo, Brazil; ^3^ Departamento de Fisioterapia, Faculdade de Medicina, Universidade de São Paulo, Sao Paulo, Brazil; ^4^ CNC-Center for Neuroscience and Cell Biology, CIBB - Centre for Innovative Biomedicine and Biotechnology, University of Coimbra, (3004-504), Coimbra, Portugal; ^5^ Interdisciplinary Program in Health Sciences, Institute of Physical Activity Sciences and Sports (ICAFE), Cruzeiro do Sul University, São Paulo, Brazil; ^6^ Polytechnic Institute of Coimbra, Applied Research Institute, Rua da Misericórdia, Lagar dos Cortiços – S. Martinho do Bispo, Coimbra, Portugal; ^7^ Hospital das Clínicas HCFMUSP (LIM52), Faculdade de Medicina, Universidade de São Paulo, São Paulo, Brazil

**Keywords:** human endogenous retrovirus (HERVs), inflammation, physiology, autoimmune diseases, aging related diseases, HERV-W, HERV-K

## Abstract

Human Endogenous Retroviruses (HERVs) are derived from ancient exogenous retroviral infections that have infected our ancestors’ germline cells, underwent endogenization process, and were passed throughout the generations by retrotransposition and hereditary transmission. HERVs comprise 8% of the human genome and are critical for several physiological activities. Yet, HERVs reactivation is involved in pathological process as cancer and autoimmune diseases. In this review, we summarize the multiple aspects of HERVs’ role within the human genome, as well as virological and molecular aspects, and their fusogenic property. We also discuss possibilities of how the HERVs are possibly transactivated and participate in modulating the inflammatory response in health conditions. An update on their role in several autoimmune, inflammatory, and aging-related diseases is also presented.

## Background on human endogenous retroviruses

**Figure 1:**
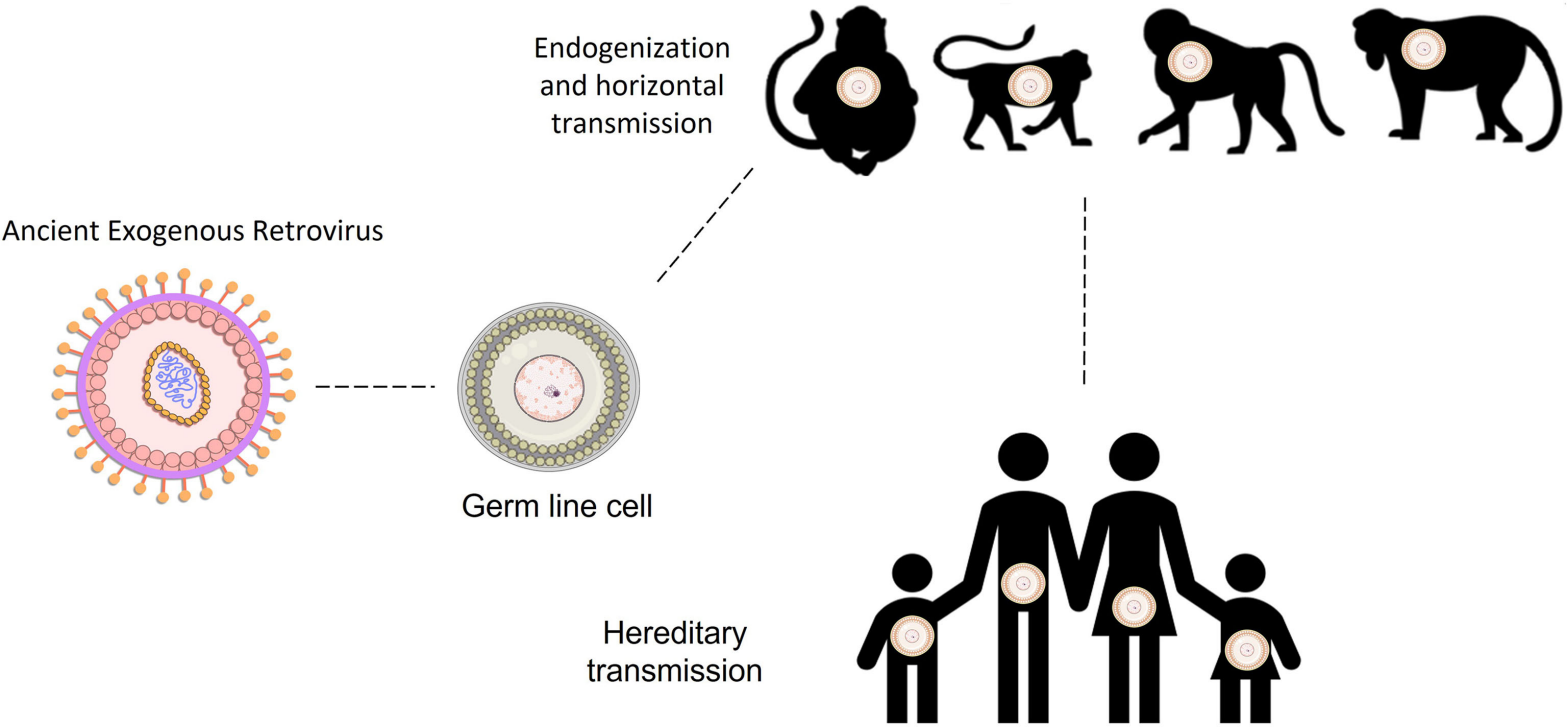
Human endogenous retroviral integration and transmission throughout host evolution of primates and humans. HERVs families were integrated into the hominid ancestral genomes in several distinct times throughout the human evolution. Draw lines between primates and human demonstrate the time between the integration moment and the present day.

Endogenous retroviruses (ERVs) were originated through ancestral retroviral infections of all vertebrates (1), by infecting their germ line cells millions of years ago. By infecting the germ line cells they were able to fix in the host genomes and be passed on through the generations by Mendelian inheritance (2–4). Due to their high transcriptional activity and replication, some time after integration they spread through reinfections, horizontal transmission, and retrotransposition (5) (**Figure 1**). More than 30 families of ERVs have been described, and several independent events of integration have occurred through human evolution (6–9). As an example, members of the family HERV-W integrated and fixed in the ancestral genomes around 40 million years ago (10–12), after the divergence between the primates of the New and Old World. Human ERVs were first described in 1970 and following human genome sequencing it was described that they make up 8% of the human genome and are therefore called Human Endogenous Retroviruses (HERV) (2–4, 13).

In terms of genomic organization, HERVs are simple retroviruses, as they present only the main genes: gag, polymerase, and envelope, flanked by two promoter regions, known as Long Terminal Repeats (LTRs) (3, 14). Briefly, the gag protein (group-specific-antigen) is genetically preserved and less immunogenic than the envelope and is responsible for the production of the viral capsid (14). The polymerase (pol) gene codes the non-structural viral enzymes, including reverse transcriptase and integrase. The envelope (env) gene synthetizes the envelope glycoproteins, and is under stronger selective pressures due to higher exposure to the host immune system (14). Finally, the LTRs are located at each end of the provirus and comprise the U3, R, and U5 regions. The U3 region contains the viral promoter and enhancer elements. The R region includes the mRNA initiation site (+1) and ends at a polyadenylation termination site (15, 16). The name of each ERV family considers the primer binding site where the reverse transcription begins (17); as an example, the HERV that presents a tryptophan as the starting site is named HERV-W (13).

**Figure 2:**
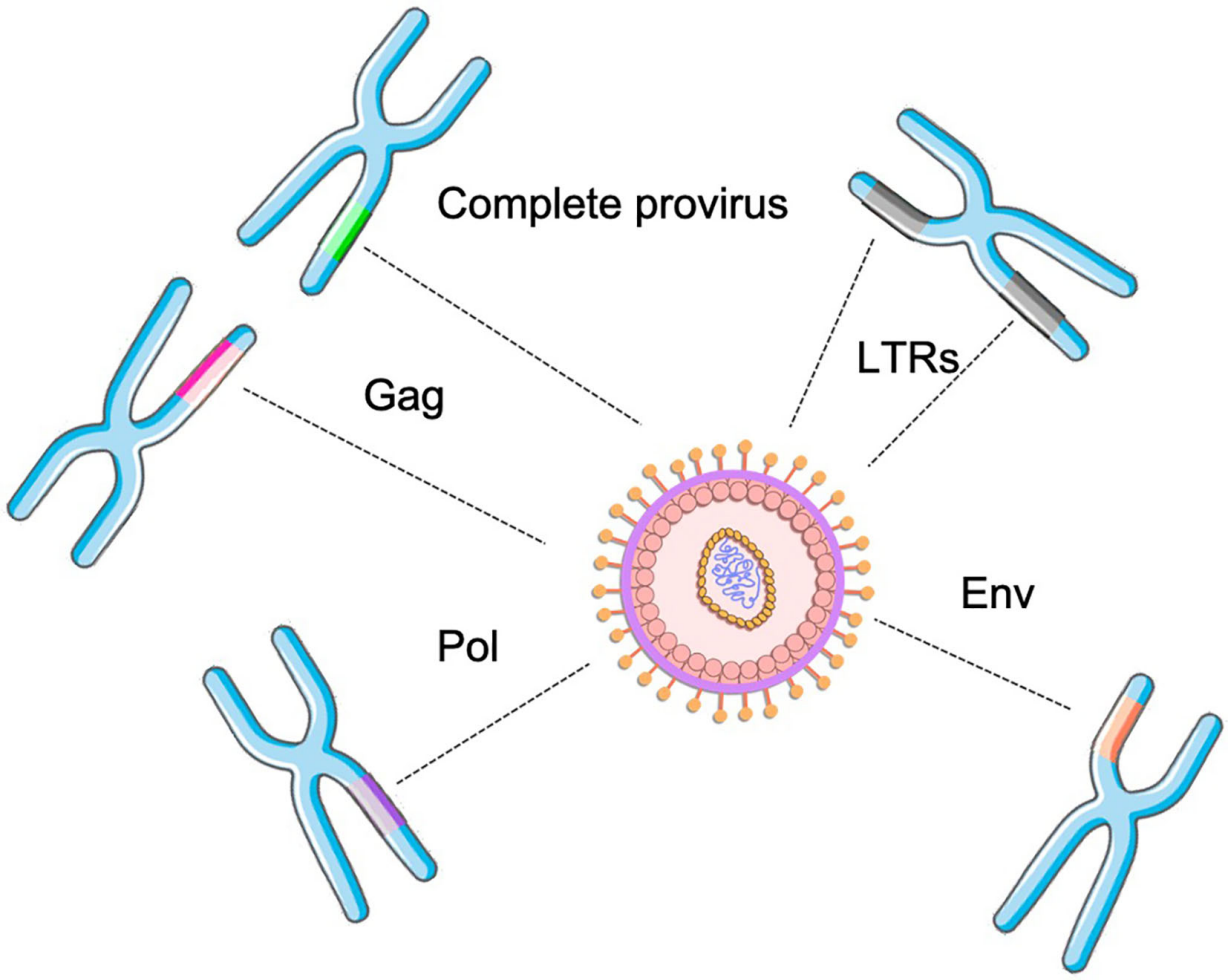
HERV assembly determined by the combination of distinct retroviral genes (solo or from complete proviruses) from different genomic locations within the genome.

Despite the high proportion of the inserted elements, there are few complete proviral sequences in the human genome. Many HERVs were purged from the host genome through evolution due to recombination, deletion, and constant mutational events. Therefore, most elements are incomplete or have deleterious mutations (e.g. isolated genes or solo LTRs throughout the genome, the presence of stop codons within proviral genes, substantial deletions, and insertions within the proviral genome). These events have led them to be unable to replicate (18). Importantly, these events have led to a widely variant distribution of HERVs within the genome. A previous study revealed that the proportion of HERV sequences within the chromosomes and population varies considerably, and also the provirus that may be expressed and to be translated into proteins (19). These findings revealed that HERVs, specially HERV-K may be widely polymorphic in human population. And in fact, HERV virions can still be found in very particular conditions, which have been extensively described for HERV-K and W (5, 20–22). Unlikely exogenous retroviruses, HERVs genes alone were distributed within the genome due to retrotransposition. Therefore, complete provirus can barely be found in human genome, culminating in the distribution of several proviral genes that still may transcribed and translated. In fact, this phenomenon can only occur by the transcription and complementation of proteins from different proviruses allocated in distinct regions of the human genome (**Figure 2**).

The acquisition and maintenance of ERVs can also have contributed to the host evolutionary processes, both at structural and regulatory levels. For instance, although most HERVs entered the host genome early in the evolution of primates, the majority of HERV families (HERV-HI, HERV-HII, ERV-9) underwent major amplification only in Old World monkeys and hominids. From a regulatory point of view, several genes are differentially expressed in humans and primates due to the presence of an LTR in the vicinity. Madstrand and Mager (23)showed the specie-specific promoter activity of a HERV-E LTR for the *apoC1* gene in humans and baboons but not in other primates.

At the structural level, some studies have demonstrated genomic rearrangements involving – and possibly driven by – different loci of HERV-K (24). The impact of the HERV-K 14C on host genomic diversity is probably among the most elegant examples. By analyzing the multiple-copy element K14C in human and primate Y chromosome, the authors demonstrated that despite the integration being present in more ancient primate hosts, the duplication event happened no longer than 10 million years ago, indicating that this retroelement contributed to the genomic diversification of this chromosome during speciation of particular primate lineages (25).

As viruses make up a considerable part of our genome and some of them are active, it is clear that these viruses may interplay within human physiology.

In this review, we will focus on discussing the main findings regarding the role of HERVs in human physiology, the mechanism that drives the inflammation, the HERV expression, and their interplay in modulating inflammatory response in both health and disease.

## Human physiology and HERVs

Regardless of how the majority of HERVs are silenced and not supposed to be expressed, many elements were fixed in the human genome and interplay with physiological activities. For instance, HERV LTRs regulate the expression of several genes (e.g. amylase, endothelin-b, pleiotrophin, apolipoprotein-C1) (23), and HERV-E LTRs serve as an enhancer for the human amylase gene (26). Syncytin-1 is one of the best-known examples of recent acquisition and domestication of a gene from an endogenous retrovirus in the human genome. This protein is encoded by an HERV-W env gene located in chromosome 7q21.2, and plays a fundamental role in the human embryogenesis. Syncytin-1 mediates the fusion of cytotrophoblasts, resulting in the formation of the placental syncytiotrophoblast (27, 28). Later in this review, we will also discuss other fusogenic roles of Syncytin-1 (29).

Similarly to Syncitin-1, Syncytin-2 is encoded by a HERV, the HERV-FRD and also participates in the placentation processes (30). The critical role of retroviral genes for many physiological activities suggests that the endogenization and fixation of retroelements was fundamental to the human evolution.

Besides the well-documented physiological roles of the HERVs, several studies point to their participation in host defense against external agents such as exogenous viruses. HIV-1 transactivates HERV-K *via* the HIV-1 tat protein, which can be expressed in TCD4+ lymphocyte and therefore TCD8+ lymphocytes may specifically respond to HERV-K antigens, selectively eliminating HIV-Infected cells (31–33). Similarly, distinct HERV families are overexpressed in tumoral tissues. For instance, the HERV-E env gene is over expressed exclusively in some types of tumors and might, through immune responses directed against retroviral proteins, eliminate tumor cells specifically. It was seen that HERV-E env peptides expressed in renal carcinoma cells are able to stimulate the TCD8+ lymphocyte response, contributing to the regression of these tumors (34, 35).

## Factors that contribute for HERVs expression

### HERV fusogenic activity in healthy and pathological conditions

Myogenesis is characterized by the growth, differentiation, and repair of cell muscle when cell fusion occurs. Cell fusion is an energy-dependent process, and the fusogens are a crucial type of membrane-bound proteins, which are mandatory for overcoming plasma membrane hybridization with associated energetic barriers (36). HERVs may act as fusogens, which was formerly described in placental tissue (27). This fusion property of Syncytin does not seem to be linked exclusively to the placentation, but also to the myogenesis and osteoclast formation. Previous findings revealed that mice that were knocked out for HERV-W (Syncytin-1) had lower muscle mass than mice that were expressing Syncytin-1, and was also related to sexual dimorphism in mice (29). In fact, Syncytin-1 may promote the fusion of myoblast (37) and ultimately the myogenesis, and may also interact with caveolin-3, which is a member of the caveolin protein that is exclusively expressed in the sarcolemma of the myocytes, and is one of the main structural proteins of the caveolae membrane in the muscle (37, 38). Importantly, strength physical exercise induces microlesions in the muscle fiber, which may result in an acute inflammatory process. Interestingly, it has been reported that inflammatory and stress conditions facilitate HERV expression (39, 40) two remarkable associations that will be discussed in the following topic. In fact, higher levels of HERV expression *in situ* were observed in high performance athletes (41). Therefore, we might envisage a scenario where the fusogenic property of HERVs, in special by HERV-W, might contribute to muscle tissue repair after strength physical exercises.

Indeed, the fusogenic property of Syncytin-1 is widely observed in the human physiology. Another noteworthy example is related to osteoclasts. These are multinucleated cells that are derived from the fusion of monocytes, and as described previously, Syncytin-1 is necessary for the fusion of these monocytes and the formation of osteoclasts. The level of expression of this endogenous retrovirus protein is higher in the plasma membrane than in other sites, and it acts in this site to induce their fusion and culminates in the formation of osteoclasts. Interestingly, Syncytin-1 also interacts with the actin filaments of the osteoclasts, which are other cytoskeletal proteins necessary to this process (42).

On the other hand, the fusogenic properties of retroviral elements may cause bi or multinucleation of cancer cells. In fact, it was reported that viruses and fusogens of human endogenous retroviral elements are a natural reservoir of fusogenic particles and proteins that could cause bi- and multinucleation of cancer cells (43). Likewise, multinucleated giant cancer cells have been found in several cancers caused by oncogenic viruses, suggesting a possible correlation between viruses and fusogens of human endogenous retroviral origin in cancer cell fusion (44).

### Oxidative stress and HERVs

Oxidative stress is physiologically described as the “imbalance between oxidants and antioxidants, in favor of oxidants, leading to disruption of the redox signaling and redox homeostasis and/or molecular damage” (45). Reactive oxygen and nitrogen species (ROS/RNS) are a group of molecules that play a role in defense and signaling, but also in damaging biological systems, depending on their rate of formation, compartmentalization (intracellular fluids, membranes, specific organelles, or extracellular space), diffusion in hydrophobic or aqueous milieu, local antioxidant defenses, etc. Spatiotemporal control of redox signaling is achieved by compartmentalized generation and removal of oxidants, which, therefore, are strongly dependent on the physicochemical and metabolic/biochemical conditions at different subcellular sites. This aspect has been conceptualized as an integrated net of (micro) redox switches that sustain cellular redox homeostasis in living cells (46) Among several ROS/RNS (and also sulfur-centered free radicals), the superoxide (O_2_•^-^) and nitric oxide radicals (NO•), together with hydrogen peroxide (H_2_O_2_) and peroxynitrite (ONOO) are the most studied signaling molecules responsible for redox homeostasis (47).

Strong evidence has shown that incorporation of a fragment or complete primal virus into the human genome, specifically an endogenous retrovirus, would have been capable of mutating the ascorbate-producing gene. The viral enzyme reverse transcriptase (RT) has been a mediator of genetic change for more than three billion years, and retroviruses have influenced the evolution of Old World monkeys and hominids (48) Interestingly, free radical-induced mutations appear to also be involved in the etiology of some cancers (49) and degenerative diseases (50). It is well-accepted that the incidence of these diseases may be a marker of the evolutionary diversification of *H. sapiens* as a species, with a greater incidence of cancer – discarding identified modern life causes, such as pollution, sedentary habits, emotional stress, anxiety, etc. – indicative of more mutations, some of which would be inheritable (51)

In agreement with the close relationship between oxidative stress and HERV expression, recent findings have associated several neurodegenerative diseases with HERV insertions into the human genome (52). The aberrant expression of HERVs is associated with neurological diseases, such as multiple sclerosis (MS) or amyotrophic lateral sclerosis (ALS), inflammatory processes, and neurodegeneration (53). HERVs are highly defective, but few complete proviruses have retained the classical genome organization of ancient retroviruses. Recent studies on multiple sclerosis (MS) demonstrated that robust oxidative stress on specific brain regions, as well as along neuro-motor circuits, were associated with upregulation of the transcription factors HERV-W/HERV-H. Moreover, HERV-W has been directly correlated with CD14 and TLR4 proteins to activate the production of proinflammatory cytokines IL-1β, IL-6, and TNF-α in affected tissues (54). The activation of TLR4 also induces NO^•^ production, aggravating the nitrosative/oxidative stress condition mediated by peroxynitrite (ONOO-) and other ROS/RNS, and thus, promoting injury to oligodendrocytes and demyelination of motor neurons (55). HERVs can also be related to amyotrophic lateral sclerosis (ALS). If HERV-K expression is forced in neurons, it causes cellular degeneration mediated by its Env protein. Transgenic mice expressing HERV-K Env in neurons developed a clinical and pathological phenotype that resembles ALS, with typical upper and lower motor neuron degeneration (56). However, what triggers the expression of HERV-K in adult neurons of patients with ALS remains unknown. *In vitro* studies showed that neuronal injury due to oxidative stress or excitotoxicity is apparently insufficient to cause activation of HERV-K genes, but there is still huge controversy about this feature (57, 58).

### Inflammatory responses and HERVs

It is broadly known that oxidative stress is closely associated with inflammation (59) and, as previously mentioned, these factors are pivotal players in the development of neurodegenerative diseases, particularly in the context of aberrant expression of HERVs (53).

Of interest, the chronic co-existence of systemic inflammation and oxidative stress (60) can also indubitably influence the activation of HERVs (52).

In terms of the inflammatory process and HERVs, there seems to be a mutual influence, in which each one can act, fueling a vicious circle between them, since it was reported that inflammation can remove the necessary blocks to limit the expression and regulation of the several genes mediated by HERV, which causes a new imbalance of gene expression, favoring an increase in instability and exacerbating the inflammatory condition (61).

In fact, it is reasonable to suggest that HERV transactivation can converge in fueling the inflammation, especially by the capacity of the HERV-W family to interact with TLRs, as formerly cited for TLR4 and CD14, leading to the induction of a prominent pro-inflammatory response, which includes the release of several cytokines, such as IL-1β, IL-6, and TNF-α (62, 63). Furthermore, it was also demonstrated that HERV can induce NF-kB activation, leading to a cytokine response involving T-helper 1 (Th1) and Th17 in a TLR2-dependent manner (64). Activation of the immune response, mainly innate immunity, by HERVs, can elicit an uncontrolled inflammation that drives the occurrence of chronic inflammation, and contributes to the development of autoimmune diseases (61), as suggested by the studies of Barrat and cols (65). and Yoshinobu and cols (66). in which self-nucleic acids, including HERVs, after detection by pattern recognition receptors (PRRs), such as TLRs, can be involved in autoantibody production in systemic lupus erythematosus.

At this point, it is paramount to highlight that, according to the results obtained in an in silico study, several transcription factors can bind within the LTR sites of HERV-K, especially those associated with NF-kB and also the interferon (IFN)-stimulated regulatory element, which increases the expression of pro-inflammatory cytokines, such as type I IFNs (61, 67). Therefore, very interesting findings demonstrate a remarkable link between the initial innate immune response, in which HERVs are pivotal elements, followed by a cellular immune response, since type 1 IFNs have essential actions in these two immune responses (61, 68)

In agreement with a previous study, TNF-α was able to increase the RNA expression of HERV-H, HERV-K, and HERV-W (69), through the TNF-α receptor signaling, that induces the activation and translocation of NF-kB for the nucleus and binds to sites presenting HERV LTRs. As specifically demonstrated for HERV-W, after TNF-α signaling, the NF-kB binds to the promoter and induces the expression of this type of HERV, mainly associated with the Env protein Syncytin-1 (70).

In a different way, it is of utmost importance to point out that in certain contexts the expression of some HERV Env proteins, such as HERV-H and HERV-FRD (Syncytin-2), could be related to an immunosuppressive action. In this respect, it is known that Syncytin-2, a HERV-FRD Env protein, presents a corollary immunosuppressive action in preventing the activation of a maternal immune response against the fetus alloantigens. Furthermore, it was reported that the immunosuppressive action of Env protein from HERV-H on the immune response in an experimental model of cancer negatively impacted the tumor cell rejection (71). Interestingly, it has been also reported that some Env proteins from HIV-1 and HERV-K can elicit the expression and release of anti-inflammatory cytokines by immune cells, mainly IL-10, through an immunosuppressive domain (isu) (72, 73), even though this modulation is variable (73) and needs to be better understood.

More recently, it was demonstrated that ERVs are also involved in both homeostatic and inflammatory responses to the microbiota (74) in a two-way relation, since exposure to microbial products was able to control the expression of ERV proteins and these proteins also drive the expression of certain microbe-derived products, particularly TLR ligands in the gut environment (75–77). As presented by Lima-Junior and collaborators (74), the level of ERV expression was crucial to control the tissue inflammatory responses to the microbiota in murine model of psoriasis, which involved exacerbated inflammation caused by *Staphylococcus epidermidis* in mice fed a high-fat diet, and also in human psoriatic lesions.

In addition to these characteristics, it also has been demonstrated that the endogenous retroelements HERV-derived can be potentially recognized by T cell receptors (TCRs) and BCRs as ‘self-peptides` and leads to immunological tolerance for them. So, the presentation of HERV `self-antigens` can favor the T and B cells to become immunologically aware of the existence of endogenous retroelements, and maybe avoid the development of some diseases, mainly autoimmune diseases. However, it was also reported that the host responses to several types of infectious agents, by increasing the transcriptional induction of endogenous retroelements, can rise the responses of T cells and B cells for these retroelements (78, 79).

Beyond these characteristics, it has been suggested that the Env protein from HERVs can act as a superantigen, able to drive polyclonal activation of lymphocytes (80), and directly impacting immune and inflammatory responses, as well as the pathogenesis of autoinflammatory diseases (81, 82). Of interest, by using quantitative PCR, it was demonstrated that the high incidence of some aging-related diseases was putatively associated with the elevation of the expression of several HERV families (83). Hence, whilst it was shown that the RNA levels of HERV-K (HML-2) and HERV-W families, in peripheral blood mononuclear cells (PBMC), can gradually increase from young to older adult individuals, it is noteworthy that due to the fact that modulation in HERV expression is under strict control, including the epigenetic aspects, such as DNA methylation, HERV expression levels between young and old individuals cannot be strikingly different. Importantly, infection by exogenous viruses, especially those whose present chronic/latent infection such as Hepatitis C virus (HCV), Herpes simplex virus type 1 (HSV-1), Human T-cell Lymphotropic Virus type 1 (HTLV-1), HIV, Esptein Barr virus (EBV), Kaposi’s Sarcoma-associated herpesvirus (KSHV), cytomegalovirus (CMV), may interfere with many of these silencing strategies and play a key role as epigenetic factors that may contributes to the HERVs activation throughout the individuals lifetime (84–90).

These pieces of information are very important and suggest that the profile of aging-dependent HERV expression can be regulated by transcriptional relaxation or restriction (82), which can impact the development of aging-related diseases, preferentially autoimmune diseases.

### HERVs and aging

Aging is a gradual process of changes that begins in early adulthood. These changes can involve some alterations, such as metabolic alterations, muscle changes, and neurocognitive decline (91).

Regardless of all the mutation accumulations, it is well-known that HERVs interact with the human genome in a positive and negative way, as discussed when considering the role of HERVs and human physiology. Importantly, with the aging-process, loss of heterochromatin and then abnormal activation retrotransposons can occur (92). The heterochromatin loss model is a fundamental genetic mechanism underlying most of the changes in gene expression observed with senescence (92). Importantly, HERV expression level in babies is low (93), but increases considerably in older adults and older people (83). Importantly, most neurodegenerative and autoimmune diseases also occur in older people (94). These results show that, although the pathogenic mechanisms are still not fully understood, the reactivation of HERVs might be associated with an increased risk of development of human aging-related diseases (95).

## HERVs expression in diseases

### HERVs and multiple sclerosis

Multiple Sclerosis (MS) is a neurological autoimmune disease that presents a progressive pattern, followed by a degenerative profile caused by continuous damage to myelin and axons (94). The disease presents a complex profile of gene expression that varies according to the clinical condition of the affected patients (96). The possible etiological role of HERVs in MS pathogenesis has been extensively studied. The first evidence dates to the late 80s, when a virus with reverse transcriptase activity was isolated from the circulating leptomeninges cells in cerebrospinal fluid (CSF) in a patient with MS. Back then, the ascension of Retrovirology was ongoing, and the researchers hypothesized that this could represent an infection event either by HIV or HTLV. However these cells tested negative for anti-p24 (HIV) and anti-p19 (HTLV) monoclonal antibodies (97, 98). Later on, the virus was cloned and characterized, its genes were identified and this new retrovirus was named Multiple Sclerosis-associated Retrovirus (MSRV), which was later found to belong to the HERV-W family (99). Since then, many studies have focused on understanding the dynamics of HERV-W transcriptional activity in MS, and it is a consensus that HERV-W expression is increased from 1.5 to 3 fold in MS patients compared with healthy controls (100–108).

Increased HERV-W activity has been repeatedly described in sclerotic plaques (106, 107), in peripheral blood mononuclear cells (100–105, 109), and in LCR (108). Regardless of the distinct clinical presentation of MS, HERV-W transcriptional activity is high (102). Many hypotheses for this increase have been raised. One feasible explanation comes from the data that MS patients present a greater HERV proviral load when compared to healthy individuals (110), and more proviruses means more mRNA. Alternatively, the generalized inflammation that MS patients are prone to present interferes with the chromatin, unsilencing the dormant HERVs. Once active, an HERV may retro-insert within other positions in the human genome, contributing to a higher HERV-W sequence load within the genome (111).

Further analysis on the HERV-W env protein, which is commonly detected in demyelinating MS brain lesions (102, 106, 112, 113), revealed that the HERV-W env protein is immunogenic, since it induces the release of inflammatory cytokines by initial agonistic effects through toll like receptor 4. This process may lead to a complex inflammatory response cascade (63, 114).

The immunopathogenic role of the HERV-W env protein has also been described *in vivo*. Perron et al., described that mice immunized with Myelin Oligodendrocyte Glycoprotein (MOG) later exposed to the HERV-W env protein developed Experimental Allergic Encephalomyelitis (EAE), one of the animal model diseases of MS. The authors also described that the clinical findings worsened according to the HERV-W-env administered doses (115). However, how this protein induces autoimmune pathological responses is still unclear. It is suggested that HERV-W might drive immune responses through molecular mimicry. In fact, HERV-W and myelin proteins, such as MOG and Myelin Basic Protein-1 (MBP-1) share at least six epitopes that could potentially cross-react (100, 116, 117).

If this can occur *in vivo*, therapeutic solutions focused on HERVs could be a possibility. In fact, Natalizumab, one of the main therapeutic strategies for MS patients, was able to reduce the levels of HERV-W expression and also the humoral response against HERV-W peptides after a few months of treatment (118, 119). Additionally, a monoclonal antibody against the HERV-W env protein was developed and shown to be safe for use in MS patients (120–124). Although the monoclonal antibody failed to reduce the acute inflammatory response, it was able prevent the neurodegenerative signs (125).

The explanation for these findings might come from the actual role of HERV-W in MS pathogenesis: Are these HERVs sufficient to trigger the immune response? or, which *loci* are key for triggering the immune response?

HERV transcriptional activity is heterogeneous and is critical to determine which proviruses are the most active in MS. Unfortunately, few studies have focused on the diversity of active proviruses. The first study devoted to understanding precisely the origin of transcripts described Xq22.3, 7q21.2, and 17q12 as the most active HERV-W loci (126). Some years later, using next generation sequencing, the previous data on the most active loci were confirmed, although no significant difference in HERV-W expression was found between MS patients and healthy individuals (127). Recently, a transcriptome analysis supported that these loci were overexpressed in MS patients, and besides HERV-W, the upregulation of other 18 HERV members was also described (109), shedding light on the broad decontrol of the HERV silencing during MS.

Although several decades have been dedicated to understanding the role of HERVs in Multiple Sclerosis pathogenesis, there are still many unanswered questions that should be considered in further studies.

### Rheumatoid arthritis and HERVs

Rheumatoid arthritis (RA) is an inflammatory disease that involves small and large joints. RA is one of the most common of autoimmune diseases and, like Multiple Sclerosis, RA is more frequently described in women than man (128). This disease is characterized by inflammation of the synovium and is linked to the destruction of articular cartilage and bone, which indicates a local immune response (129). HERVs from distinct families seem to be overexpressed in RA patients, such as HERV-K, HERV-L, HERV-W, and ERV-9 (130, 131). Although less explored than in Multiple Sclerosis, some studies point to a relation between HERV expression and RA.

Importantly, both the number of RA patients who present antibodies against the HERV-W env protein and antibody titer levels are higher than in healthy subjects (132), indicating that the humoral response against HERV is upregulated in the disease. Contrasting results, however, reported that the protein HERV-K rec, analog to HIV rev protein, was detected in the synovial tissue of both healthy and RA patients, and it was actually down regulated in RA. Interestingly, HERV-K rec proteins derived from an alternatively spliced gene were detected in the synovia of RA patients (133).

A genetic association between RA patients and HERV LTR has also been postulated, since polymorphisms were detected in HLA-DBQ81 alleles, where HERV LTRs are present. Importantly, these genetic alterations are related to deletions or the presence of distinct LTRs within these alleles and reveal a diversity of LTR profiles that might be linked to RA (134).

### HERVs and COVID-19

Severe acute respiratory syndrome coronavirus 2 (SARS-CoV-2) emerged in late 2019 in China, causing a global pandemic of a disease later named Coronavirus Disease 2019, or COVID-19. Most patients with COVID-19 will experience mild or moderate respiratory symptoms, while severe COVID-19 is characterized by intensive inflammatory responses, and high viral loads, and affects mostly older adults and individuals with comorbidities. Better understanding of viral immunopathogenesis is needed to identify new avenues for treatments (135).

Research relating HERVs to COVID-19 is ongoing and until now, accumulated data indicate differential expression of some HERV families during the acute phase of the disease, generally associated with enhancement of inflammatory processes (136, 137). It was initially described that the envelope genes from HERV-W and HERV-K were highly expressed in peripheral blood mononuclear cells (PBMC) from healthy individuals exposed to SARS-CoV-2 *in vitro*, but only the HERV-W-env protein was synthesized. Subsequently, *in vivo* analysis confirmed an increased level of HERV-W-env in COVID-19 patients. The authors pointed out that HERV overexpression was apparently a consequence of direct transactivation by the presence of SARS-CoV-2 and not a consequence of the inflammatory process experienced by the cytokines and chemokines released during the infection (136, 137).

As COVID-19 has different manifestations, from asymptomatic to the most severe form, which usually affects older adults and people with comorbidities, studies have been carried out to understand whether the elevated expression of HERV contributes to the worsening of the disease. In Balestrieri et al. (2021) (136) a higher percentage of the HERV-W-env protein was seen in leukocytes, especially in TCD3+ lymphocytes of COVID-19 patients, which was also correlated with the expression of programmed cell death 1 (PD1), an exhaustion marker, and in CD8+ T cells, with the expression of CD57, a senescence marker. This correlation was also associated with the COVID-19 severity and reflected the respiratory outcome of the patients during hospitalization (136).

These data were later supported by another group that evaluated HERV expression of the human bronchial epithelial cell lineage (HBEC) with induced senescence. The authors described overexpression of distinct HERV families, including HERV-K, HERV-W, and HERV-FRD, this last one being the most upregulated element among them, in comparison with the noninduced HBEC (138). In this work, the authors also found complete HERV dysregulation in bronchoalveolar lavage fluid (BALF), but not in PBMC, strongly suggesting a role of HERVs in the inflammatory process. Similarly, Marston et al. (2021) demonstrated upregulation of retroelements in BALF but not in PBMC. The authors claim that such a different profile of HERV expression could be explained by differences in permissibility to viral infection of blood cells with regard to the cells present in BALF (138, 139).

The studies performed in COVID-19 patients are not limited to mRNA detection. As already observed in autoimmune diseases, Simula et al. (2022) described the presence of anti-HERV-W-env and anti-INF I antibodies in ICU COVID-19 patients (140), suggesting that the inflammatory component of severe COVID-19 may be related to the presence of HERV antigens.

In agreement with the hypothesis that HERV overexpression is related to disease severity, Temezoro et al. (2022) described the upregulation of HERV-K genes in tracheal aspirates from COVID-19 patients submitted to invasive mechanical ventilation (IMV) and the overexpression of this retroelement was also associated with early mortality (141). Guo et al. (2022) used real time PCR to investigate if the expression of HERV-K (HML-2) could stimulate the synthesis of IFN-1 in patients with COVID-19. The authors found that gag, pol, and env HERV-K genes were highly expressed *in vivo* and *in vitro* through SARS-COV-2 infection and were positively correlated with IFN-related gene expression in moderate and severe cases (142). The authors, however, associated the HERV-K upregulation with a protective role, since as more HERV mRNA was detected more INF genes were activated.

A study performed by Tovo et al. (2021) also investigated HERV activity and the expression of genes related to the antiviral responses in COVID-19 children, but described slightly different results. The authors observed that proviral genes from HERV-H, HERV-K, and SYN-1 and SYN-2 were upregulated in mild and moderate cases of COVID-19, but not in severe cases. They also found positive correlations between TRIM28/SETDB1 and HERV activity, suggesting that the upregulation of HERVs in mild and moderate cases was able to enhance the innate protective mechanisms. The distinct HERV profile seen in children and adults supports the hypothesis that increased expression of HERVs could be associated with a more severe form of the disease in people with advanced age, but not in children (143).

In summary, from the data accumulated to date from SARS-COV-2 and HERVs, it is only possible to associate HERV dysregulation with COVID-19 severity. The significance and mechanisms involved in the HERVomics remain to be elucidated.

### Systemic lupus erythematosus and HERVs

Systemic lupus erythematosus (SLE) is a complex multisystem and autoimmune disorder that affects predominantly women of childbearing age (144). Lupus was initially described as a dermatological condition but it is currently known that the clinical features of SLE are much wider and any system can be affected. Some of the most common symptoms may include fatigue, thrombocytopenia, skin rash, arthritis and arthralgia, and glomerulonephritis. Genetic and environmental factors appear to contribute to the pathogenesis of SLE, although an inheritance concordance rate is only moderate in both monozygotic and dizygotic twins. Women comprise 90% of SLE cases, and hormones are recognized as contributors to SLE development, since estrogen and prolactin enhance immune responses through diverse mechanisms (145). The production of anti-nuclear antibodies (ANAs) is a hallmark of SLE, so their detection in the blood of suspected patients is considered a confirming diagnostic (146).

Infections have been implicated in SLE development either as a causal or protective role for many years. While Epstein–Barr virus and Cytomegalovirus are considered as putative triggers, other infectious agents such as HBV are believed to play a protective role (147, 148). Direct and indirect evidence of a link between retroviral infections and SLE etiology has also been demonstrated (149). The evidence ranges from the detection of interferon production to the presence of antibodies anti- HIV-1 p24 and p17 gag proteins in SLE patients (150, 151). More recently, however, PCR tests failed to demonstrate the presence of HIV or any other exogenous retroviruses in these patients (152). Coincidently, the discovery of HERVs has helped to solve the puzzling findings.

Several authors have described the increased detection of HERV mRNA in SLE individuals, particularly he HERV-K102 (153), HRES (154), and HERV-E 4-1 (155, 156). A broader analysis based on RNA sequencing found six families of HERVs differentially expressed in SLE; the ERV-L, ERV3, MER4, HERV-H, HERV-K, and HERV-L families. Interestingly, many of the overexpressed elements were located near genes related to the immune system or the innate immune response to viruses, consistent with the involvement of innate immunity in SLE (157).

The belief in participation of retroviruses in the pathogenesis of SLE also arose through a series of SLE mouse NZBxNZW/F1 models. Elevated serum levels of a retroviral glycoprotein gp70 were demonstrated as well as high levels of interferon, as seen in patients with SLE (158).

Besides mRNA detection in humans, antibodies and cellular responses directed to HERVs have also been described, although the immune response against HERV-derived proteins is hard to explain since, as self-antigens, they should not induce immune responses. However, using recombinant proteins, Perl and colleagues demonstrated that almost half of patients with some autoimmune disease and 52% of those with SLE presented antibodies against HRES-1 (159). A few years later, a Japanese study also described that a significant proportion of SLE patients presented antibodies against gag proteins from HRES-4-1, and some of them against env proteins (160). Polymorphic genotypes of HRES-1 are also correlated with SLE. Magistrelli et al. (161) demonstrated that a polymorphic HindIII site, identified as a G/C transition at position 653 of the long terminal repeat region, defines two allelic forms of the HRES-1 genomic locus, which are differently found in SLE and non-SLE individuals. To date, the weight of evidence put HRES-1 among the best ERV candidates for participating in SLE etiopathology.

Although the HERVs overexpression in autoimmune diseases is well recognized, the link between the retroelements activation and autoimmunity is still controversial.

In fact, the reduced expression of epigenetic repressor genes due to global genomic hypomethylation observed in SLE (153, 162) may well explain the overexpression of several originally silenced endogenous retroviruses and retroelements. However, despite the myriad of retroelements and ERV genomes scattered through the human genome, only specific elements/families are unsilenced during the epigenetic dysregulation that occurs in SLE. For instance, although HERV-E clone 4–1 upregulation is mostly due to the hypomethylation at LTR2C in lupus CD4+ T cells, it is supposed to participate in disease pathogenesis *via* miR-302d/MBD2/DNA hypomethylation and IL-17 signaling through the 3’LTR (156).

Even if the overexpression of retroelements is not sufficient to trigger the disease, this does not mean that it is not detrimental, since their expression can somehow elicit immunological responses. The simple accumulation of HERV-derived nucleic acids can stimulate interferon and anti-DNA antibody production in SLE. In fact, HERVs are believed to be implicated in SLE pathogenesis in different ways, which include structural or functional molecular mimicry, innate immune activation through IFN production, promoting or enhancing the transcription of neighbor genes, and by superantigen production (116, 163, 164). The HERV expression itself is sufficient to activate cells of the immune system, especially CD4 T lymphocytes, since they encode proteins that can act as superantigens (165).

Molecular mimicry between endogenous and retroviral proteins has long been suggested to explain atypical immunological responses found in patients suffering from distinct autoimmune disorders (161). Autoantibodies to nuclear proteins are associated with immune complex formation and tissue deposition, as observed in murine models and in humans for HERV-K102. These complexes tend to recruit and activate inflammatory cells (153, 158). In addition, the autoantibodies that recognize snRNPs have been described in some rheumatic diseases, including SLE, and this protein contains a region that cross reacts with a conserved domain within the Gag protein of mammalian-type C retroviruses (166), such as the HRES. Talal and colleagues (150) described that anti-Sm antibodies, a hallmark in SLE, also partially cross-react with p24 gag. It was not difficult to conclude that the presence of anti-HIV antibodies in SLE patients in the absence of HIV infection can only be explained through cross-reactivity with endogenous proteins/antigens, likely produced by retroelements (167, 168).

Although the true causative role of HERVs in autoimmune diseases remains to be proved, their involvement in SLE pathogenesis in different degrees is well demonstrated.

In summary, HERV expression in the host genome is influenced by external (UV light, infectious agents, and chemical elements), internal (hormones chemokines and cytokines), and epigenetic factors (such as DNA methylation and histone modification) (169). Although the overexpression of HERVs and other retroelements in several pathological and autoimmune diseases is recognized, there is little mechanistic understanding of how HERVs contribute to local or systemic inflammation. It is also a matter of debate whether the overexpression is a causative agent of the autoimmunity or a simple bystander.

## Author contributions

All authors contributed to the article and approved the submitted version.

## Funding

Fundação de Amparo a pesquisa do Estado de São Paulo (FAPESP) Grant #2013/24223-9 and #2015/05958-3

## Conflict of interest

The authors declare that the research was conducted in the absence of any commercial or financial relationships that could be construed as a potential conflict of interest.

## Publisher’s note

All claims expressed in this article are solely those of the authors and do not necessarily represent those of their affiliated organizations, or those of the publisher, the editors and the reviewers. Any product that may be evaluated in this article, or claim that may be made by its manufacturer, is not guaranteed or endorsed by the publisher.
